# Prevalence and Types of Inappropriate Antibiotics Prescribing Among Dialysis Patients: A Systematic Review

**DOI:** 10.3390/antibiotics14101049

**Published:** 2025-10-20

**Authors:** Sara Abul-Ola, Reem Alenany, Usman Abubakar

**Affiliations:** 1 College of Pharmacy, QU Health, Qatar University, Doha P.O. Box 2713, Qatar; sa1801871@student.qu.edu.qa (S.A.-O.); ra1702907@student.qu.edu.qa (R.A.); 2 Department of Clinical Pharmacy and Practice, College of Pharmacy, QU Health, Qatar University, Doha P.O. Box 2713, Qatar

**Keywords:** renal replacement therapy, antibiotics, prescribing patterns, appropriateness of antibiotic prescribing

## Abstract

**Background/Objectives**: Understanding the patterns of inappropriate antibiotic prescribing is crucial to design antimicrobial stewardship interventions. This systematic review evaluated the prevalence and types of inappropriate antibiotic prescribing among dialysis patients. **Methods**: Four electronic bibliographic databases including PubMed, Embase, Scopus, and CINAHL, were searched. Supplementary search was conducted using Google Scholar and by manually checking the reference list of selected studies. Selected studies include those published in the English language since inception of the databases until October 2024. Two independent reviewers screened, selected, and extracted the data for qualitative synthesis. **Results**: Of the 784 records identified from the databases, 13 studies fulfilled the eligibility criteria. Eight of the studies (42.6%) were from the USA. Antibiotic prescribing rate ranging from 16 to 75.5% was reported among dialysis patients, with vancomycin (6.5–100%), piperacillin-tazobactam (2.4–44.5%), meropenem (2.1–25.8%), metronidazole (2.1–16.4%), cefazolin (4.3–13.6%), and ceftriaxone (1.3–10.8%) being the most commonly prescribed antibiotics. The studies showed that 20–65.7% of prescriptions are inappropriate, mostly due to inappropriate dosing (25.5–100%), lack of an indication (5.5–73.9%), and inappropriate choice/spectrum (23.6–69.7%). **Conclusions**: Antibiotic prescribing among dialysis population is higher than the rate reported among hospitalized patients. High rate of broad-spectrum antibiotic prescribing coupled with the high rate of inappropriate antibiotic prescribing indicate the need for the implementation of antimicrobial stewardship programs in dialysis settings.

## 1. Introduction

The burden of chronic kidney disease (CKD) is a growing global health concern, affecting approximately 10% of the world’s population. The progression of CKD leads to end-stage renal disease (ESRD), where renal replacement therapy in the form of either dialysis or transplantation would be required. The median prevalence of ESRD patients treated with maintenance dialysis is 823 per million population, globally [1,2,3]. Patients with advanced CKD are at increased risk of all-cause mortality and morbidity, frequently necessitating hospitalization. Approximately 50% of mortality among CKD patients is attributed to non-cardiovascular causes, with infection being a leading factor. Dialysis patients experience infections at a rate of 5.7 events per 1000 dialysis days [4,5]. These patients are particularly vulnerable to infections caused by multidrug-resistant organisms (MDROs), with colonization and infection rates often exceeding those seen in other populations [6]. Antimicrobial use, along with patient-to-patient transmission of resistant strains, has caused a rapid increase in the prevalence of antimicrobial resistance (AMR) which has rapidly emerged as a global public health threat in the 21st century, endangering the prevention and treatment of various infections caused by microorganisms no longer susceptible to conventional, once-effective antimicrobials [7,8]. Antimicrobials overuse and misuse have been associated with AMR explosion, thus highlighting the critical need to optimize antimicrobial use in this vulnerable population [9,10]. Infections caused by MDROs are associated with high rate of mortality [11].

Unfortunately, the appropriate use of antibiotics in dialysis patients presents significant challenges due to altered pharmacokinetics and pharmacodynamics caused by impaired renal function and renal replacement therapies. These alterations affect drug clearance and can lead to both sub- and supra-optimal dosing, increasing the risk of treatment failure, toxicity, and the emergence of MDROs [12,13]. Moreover, studies have shown that up to 30% of antibiotic doses administered in outpatient dialysis settings may be inappropriate [10,14], with errors in dosing, duration, or indication contributing to unnecessary antibiotics exposure and resistance [14,15]. Addressing these inappropriate prescribing practices is critical to reducing the burden of MDROs and improving patient outcomes. Given the complex interplay between infection risk, antibiotics use, and resistance in the dialysis population, a systematic review summarizing existing evidence on the appropriateness of antibiotic use in these patients is essential. Such a review will provide clinicians with a comprehensive understanding of current prescribing patterns and identify gaps in knowledge. The findings could inform the development of targeted antimicrobial stewardship interventions, particularly in dialysis settings where inappropriate antibiotics prescribing is prevalent [10,14]. The primary objective of this review is to assess the rate and types of antibiotics prescribed among patients undergoing dialysis across different healthcare settings. The secondary objective is to evaluate the prevalence and types of inappropriate antibiotics prescribing in patients undergoing dialysis.

## 2. Results

### 2.1. Search Results and Study Selection

**Figure 1:**
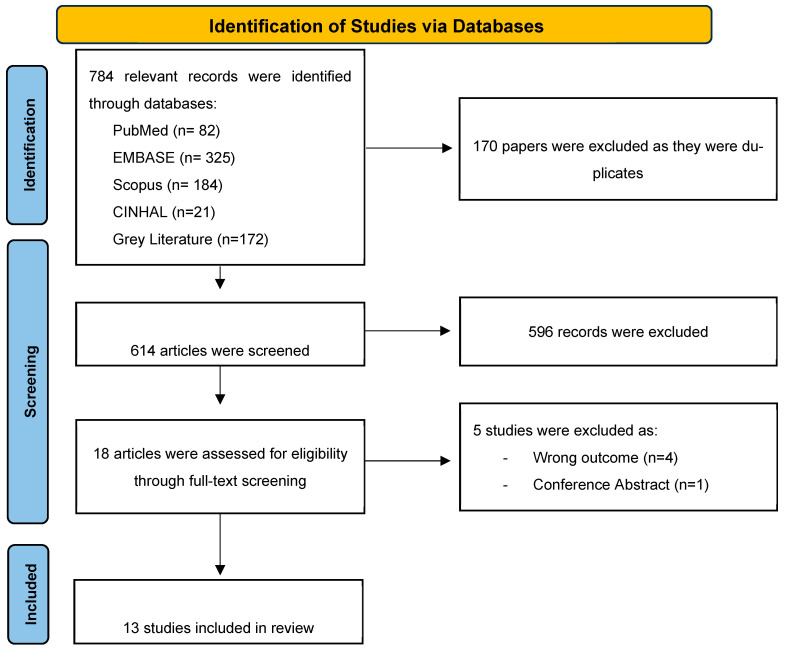
PRISMA flow diagram for studies evaluating antibiotic use and/or appropriateness in dialysis patients.

Of the 784 relevant records identified from databases and reference screening, 170 duplicate articles were removed. An additional 596 articles were excluded after title and abstract screening for being irrelevant, non-clinical studies, conference abstracts, or surveys. This resulted in 18 articles for full-text screening, out of which 12 studies were included in this systematic review. An additional study was identified through manual search of the reference lists. Figure 1 describes the articles’ identification, screening, and selection process.

### 2.2. Study Characteristics

Ten studies (77%) were conducted in North America, including eight from the USA [16,17,18,19,20,21,22,23] and two from Canada [24,25]. The remaining studies are from Oman (7.7%) [26], Australia (7.7%) [15], and India (7.7%) [27]. Most studies (69.2%) used retrospective data [16,18,19,20,21,22,24,25,26], while three studies (23%) used prospective data [15,23,27]. The studies included 20,568 dialysis patients with individual study population ranging from 42 to 18,402 patients. Hemodialysis (84.6%) was the most common dialysis modality, and continuous replacement therapy “CRRT” was used in five studies (38.46%). The mean age of the patients ranged from 49 to 66.7 years. Table 1 summarizes the characteristics of the studies included in the review.

### 2.3. Quality Assessments of Included Studies

**Figure 2:**
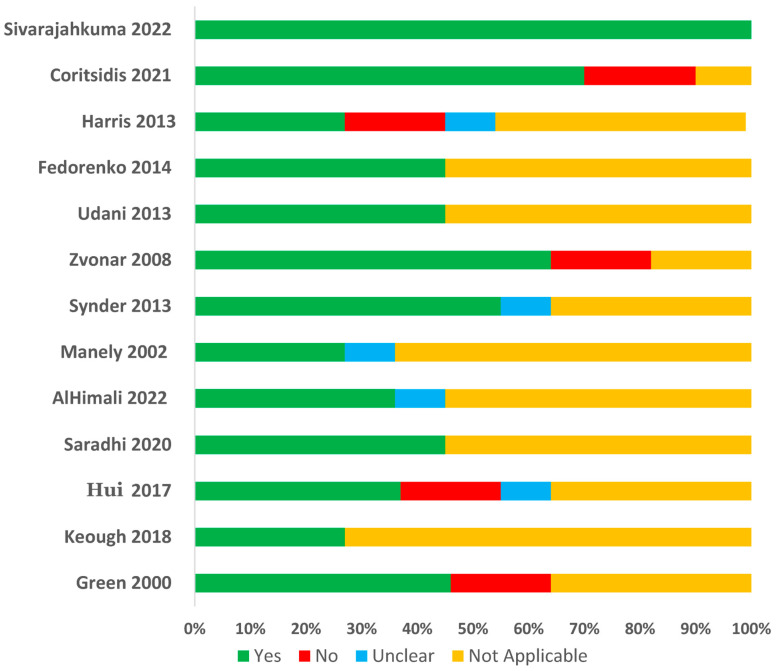
Quality assessment of included studies [15,16,17,18,19,20,21,22,23,24,25,26,27].

Eleven studies were assessed using the cohort study checklist, with ten of them measuring the exposure and outcomes in a valid and reliable way [16,17,18,19,20,21,23,24,25,26]. Nine studies used appropriate statistical methods for analysis [15,16,17,18,19,21,23,25,26], while two studies were adjudged to have inappropriate statistical analysis [20,25]. Case series checklist was applied for one study and the study received a score of ‘yes’ on all items [24], while the case-control checklist was applied for one study which was rated ‘yes’ in six out of ten items [22]. Figure 2 summarizes the quality assessment results for the 13 included studies. For each quality criterion, a study receives a “Yes” (shown in green) if it meets the criteria, a “No” (shown in red) if it does not, “Unclear” (shown in blue) if the information is not clearly mentioned, and “Not Applicable” (shown in yellow) if the study’s design or methodology is not related to the question.

### 2.4. Prevalence and Types of Antibiotics Used Among Dialysis Patients

**Figure 3:**
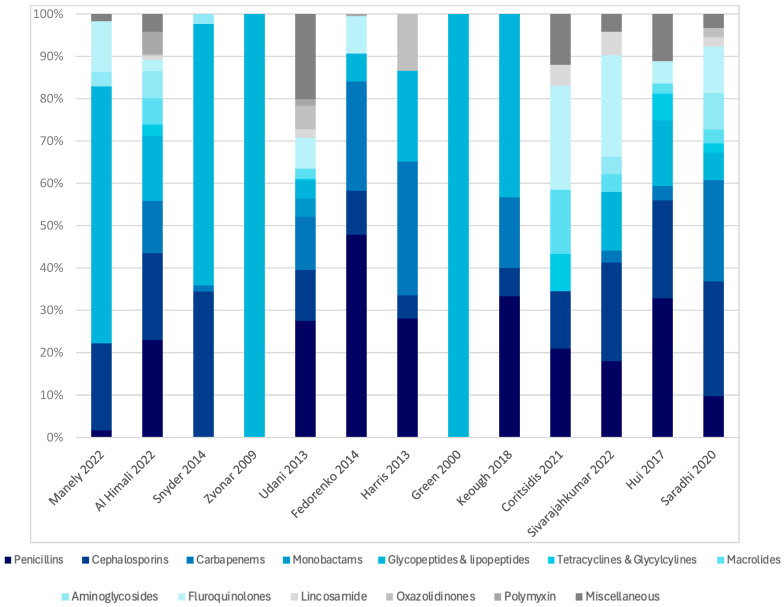
Distribution of the classes of antibiotics prescribed among dialysis patients [15,16,17,18,19,20,21,22,23,24,25,26,27].

The studies involved 55,667 antibiotics prescribed to the patients in the selected studies. The rate of antibiotic use, defined as the proportion of patients on dialysis receiving at least one antibiotic during the study period, was reported in eight studies (61.5%) and ranged from 16% to 75.5% [15,16,18,19,21,23,24,27]. Antibiotics were mostly prescribed for empirical therapy (7.6–100%), definitive therapy (11–86.2%), and prophylaxis (0.6–11%). Bloodstream and respiratory tract infections are the most common infections treated with antibiotics. Glycopeptides and lipopeptides are the most common classes of antibiotics prescribed among the patients, with rates ranging from 6.5 to 100%, followed by penicillins (1.7–47.8%), cephalosporins (5.5–35.1%), carbapenems (1.5–31.6%), fluoroquinolones (2.6–23%), aminoglycosides (3.4–8.7%), and polymyxins (0.5–5.2%). Figure 3 illustrates the classes of antibiotics prescribed among the patients. Vancomycin (6.5–100%), piperacillin-tazobactam (2.4–44.5%), meropenem (2.1–25.8%), metronidazole (2.1–16.4%), cefazolin (4.3–13.6%), ceftriaxone (1.3–10.8%), ciprofloxacin (1.9–10.3%), and colistin (0.5–5.2%) were the most commonly prescribed antibiotics.

### 2.5. Appropriateness of Antibiotics Prescribed Among Dialysis Patients

Ten studies (77%) assessed the appropriateness of antibiotics prescribed among dialysis patients [15,17,18,19,20,21,23,25,26,27]. International guidelines were the most common tool used to evaluate the appropriateness of antibiotic prescriptions (*n* = 6/11; 54.5%), followed by national guidelines (18.2%), and literature-based recommendations (18.2%). The rate of inappropriate antibiotic prescribing reported ranged from 20 to 65.7% [15,17,18,19,20,21,23,25,26,27]. The most common types of inappropriate antibiotic prescribing included inappropriate dosing (25.5–100%) [15,18,19,20,21,26,27], lack of indication (5.5–73.9%) [15,17,23], inappropriate choice/spectrum (23.6–69.7%) [15,17,23,25], inappropriate interval (8.7–57.6%) [15,18,21,26,27], and inappropriate duration (10.9%) [15]. A review of the data stratified by dialysis modality highlights important distinctions in dosing inappropriateness. Studies focusing on continuous or hybrid modalities reported particularly high rates of dosing errors. For example, one study evaluating patients on continuous venovenous hemodialysis (CVVHD) found that 100% of inappropriate prescriptions were due to dosing errors [21]. Similarly, studies on patients receiving sustained low-efficiency dialysis (SLED) reported that 100% and 85% of inappropriate prescriptions, respectively, were related to incorrect dosing [22,23]. Although dosing errors were also notable in studies of intermittent hemodialysis (IHD), the rates were more variable, ranging from 25.5% [15] to 91.3% [28].

Table 2 shows the rates of inappropriate antibiotics prescribing reported in the included studies.

## 3. Discussion

This systematic review evaluated the prevalence and types of antibiotics used among dialysis patients, as well as the rates and types of inappropriate antibiotic use. This review aimed at identifying antimicrobial stewardship opportunities to improve quality use of antibiotics, improve clinical outcomes, and reduce adverse effects, resistance, and healthcare costs among dialysis patients. There is a lack of studies investigating antibiotic use among dialysis patients from Africa, Europe, and South America. The included studies utilized different study designs including retrospective and prospective cohort study design, case-control design, and case-series. The current review found that the rate of antibiotic use among dialysis patients varies between the studies, ranging from 16% to 75.5%. This variation is attributed to the differences in antibiotic use between inpatient and outpatient dialysis settings, with inpatients typically receiving antibiotics for acute or more severe infections, while outpatients are generally prescribed antibiotics for chronic or less severe conditions. Antibiotic use in dialysis population is higher than the rate reported among maintenance hemodialysis patients [29], and hospitalized patients in Europe (30.5%) [28] and the United States (49.9%) [30]. In addition, antibiotic prescribing in dialysis settings is higher than the rate reported in the emergency department [31]. High rate of antibiotic use in dialysis population could be explained by their susceptibility to infectious complications [29,32], mainly due to their impaired immunity, regular vascular access, and multiple comorbidities [33,34]. This finding is corroborated by the fact that chronic dialysis patients with a history of colonization or infection with multidrug-resistant organisms have an increased risk of receiving antibiotics [35]. In addition, tunneled catheter access and daytime dialysis sessions have been associated with antimicrobial use [35]. Furthermore, there is limited evidence describing the implementation of antimicrobial stewardship programs in dialysis settings to reduce unnecessary and excessive use of antibiotics. Therefore, strict compliance with infection prevention and control strategies, and antimicrobial stewardship interventions are recommended to reduce infectious complications and reduce the excessive use of antibiotics in the dialysis population [29].

Broad-spectrum antibiotics including those categorized as “Watch group antibiotics” such as vancomycin, piperacillin-tazobactam, meropenem, ceftriaxone, and ciprofloxacin, and the “Reserve group antibiotics” such as colistin and linezolid are the most common antibiotics used among dialysis patients. The most common “Access group antibiotics” used among dialysis population include cefazolin and metronidazole. Watch group antibiotics generally exhibit a high propensity for the emergence of antibiotic resistance and have been recognized as potential targets for monitoring and for antimicrobial stewardship interventions [36,37]. Reserve group antibiotics are usually reserved for the management of critical/high-priority multidrug-resistant bacterial infections [36,37]. Previous studies have shown relatively high rates of broad-spectrum antibiotic use among dialysis patients [29], and among hospitalized patients in general [28,30,38]. In addition, studies have shown increased risk of multidrug-resistant infections/colonizations among dialysis patients [39,40,41], and this could explain the high rate of broad-spectrum antibiotic use. Furthermore, bloodstream infections, vascular access infections, and sepsis are the most common infections reported among dialysis patients [12,33,34]. These infections require empirical treatment with broad-spectrum antibiotics to reduce morbidity and mortality [42]. Nevertheless, these findings highlight the need for antimicrobial stewardship program in dialysis population to ensure rational use of these delicate groups of antibiotics to preserve their effectiveness and prevent the emergence of antibiotic resistance.

Inappropriate use of antibiotics has been recognized as one of the major drivers of antibiotic resistance [29,32,37], and antibiotic stewardship interventions are aimed at promoting rational use of antibiotics to prevent resistance [29]. The current study found that inappropriate use of antibiotics is relatively high among dialysis patients, with 20–65.7% of antibiotic prescriptions adjudged to be inappropriate. This is higher than the rate of inappropriate antibiotic prescribing reported among hospitalized patients (about one-third) [43], and among outpatients (approximately 50%) [44]. This could be explained by the high rate of antibiotic use and the lack of antimicrobial stewardship program in dialysis settings. Therefore, the high rate of antibiotic use coupled with the high rate of inappropriate antibiotic use clearly underlines the need for the development and implementation of antimicrobial stewardship programs in dialysis settings.

Understanding the types of inappropriate antibiotic use is crucial for the design of effective antimicrobial stewardship interventions. Inappropriate dosing, including under-dosing and overdosing of antibiotics, was the most common type of inappropriate therapy among dialysis patients. A previous study revealed that 58% of parenteral antibiotics used among outpatient dialysis population are inappropriate, and this was significantly associated with the tunneled catheter access and the duration of chronic hemodialysis [35]. The existing literature acknowledged the pharmacokinetics and pharmacodynamics alterations of antimicrobial agents in patients receiving renal replacement therapy [45,46,47]. Therefore, antimicrobial dose adjustment and optimization is required for dialysis patients. A previous study demonstrated the positive effect of pharmacists’ participation in antimicrobial dose adjustment among critically ill patients receiving continuous venovenous hemofiltration, including a reduction in costs and adverse drug effects [48]. This finding denotes the important role of clinical pharmacists in dose adjustment and optimization in antimicrobial stewardship programs [49]. However, a lack of research competence and limited antimicrobial stewardship knowledge have hindered pharmacists’ effective participation in antimicrobial stewardship programs [49,50].

Another form of inappropriate prescribing identified was antibiotic use without an indication among dialysis patients. This involves empirical use of antibiotics in patients who did not meet standard diagnostic criteria for infections, surgical prophylaxis without an indication, and the failure to stop empiric antibiotics in patients with negative culture results, consistent with the result of a previous study [29]. Furthermore, the current study also revealed a high rate of inappropriate choice/antibiotic spectrum among dialysis patients, similar to a previous study [29]. The failure of nephrologists to streamline empirical antibiotics therapy based on culture results has been acknowledged in the literature, including failure to de-escalate from vancomycin to beta-lactams in patients with bloodstream infection due to methicillin susceptible *Staphylococcus aureus* (MSSA) [29]. Evidence has shown that beta-lactams are superior to vancomycin for the treatment of MSSA bacteremia [51,52]. The inappropriate use of antibiotics among dialysis patients coupled with the high rate of antibiotics with greater propensity for resistance highlights the urgent need for the implementation of antimicrobial stewardship programs in dialysis settings [29,32]. Few studies have demonstrated the positive impact of antimicrobial stewardship programs on antibiotic prescribing and patient outcomes in this population [53,54]. Antimicrobial stewardship interventions recommended include educational training for prescribers, prospective/retrospective review with feedback, dose optimization and adjustment, therapeutic drug monitoring and antimicrobial ward rounds. However, it is important to understand local prescribing and common inappropriate prescribing patterns to deliver effective interventions to improve practice.

This review has a number of limitations that should be considered while interpreting the findings. First, there is a lack of studies from Africa, Europe, and Latin America, and this limits the generalizability of the results. This could be attributed to the language bias in the search for studies included in this review. Second, there is heterogeneity in the assessment of inappropriate antibiotics prescribing, with some studies using international guidelines while others used national guidelines or literature-based recommendations. These inconsistencies introduce an assessment bias that ought to be considered while interpreting the study findings. Third, extracted data was synthesized qualitatively without a meta-analysis and this is justified based on the variations in the tools used to assess appropriateness of antibiotic prescriptions in dialysis population. Fourth, the included studies used different study designs, which affected the assessment of methodological quality. Fifth, some of the included studies involved acute dialysis patients without end-stage renal disease such as acute kidney injury patients. Despite these limitations, this systematic review provides a comprehensive summary of antibiotics prescribing, and the rate and patterns of inappropriate antibiotic prescribing among dialysis patients.

## 4. Materials and Methods

### 4.1. Study Design

This is a systematic review of published studies that was conducted in accordance with the Preferred Reporting Items for Systematic Reviews and Meta-analyses (PRISMA) 2020 guidelines [55]. The study protocol was registered on PROSPERO with the following reference number CRD42025637423.

### 4.2. Eligibility Criteria

#### 4.2.1. Inclusion Criteria

- Primary peer-reviewed clinical studies assessing antibiotics prescribing patterns (dosing, indication, route, duration, spectrum) and their appropriateness in dialysis patients.
- Observational studies (both retrospective and prospective) published in the English language.
- Studies that included adult patients (aged 18 years and above) with CKD undergoing chronic or acute dialysis (e.g., intermittent HD “IHD”, continuous renal replacement therapy “CCRT”, peritoneal dialysis “PD”, Prolonged Intermittent Renal Replacement Therapy “PIRRT”).
- Studies conducted in inpatient and outpatient settings, including tertiary hospitals, community healthcare centers, dialysis units, etc.
- No time restriction for studies, with inclusion up to October 2024.

#### 4.2.2. Exclusion Criteria

- Studies focusing on non-clinical participants such as animal models or laboratory-based pharmacokinetics.
- Conference abstracts, editorial commentaries, reviews, case reports, and duplicate studies.
- Unpublished reports, or articles from non-peer-reviewed sources.
- Studies involving exclusively non-dialysis CKD patients, patients with acute kidney injury (AKI), or pediatric population.

### 4.3. Information Sources

Electronic databases (PubMed, Embase (Elsevier, Amsterdam, The Netherlands), Scopus (Elsevier, Amsterdam, The Netherlands), CINAHL (EBSCO Information Services, Ipswich, MA, USA)), references of selected articles, and references of other scoping or systematic reviews were searched to identify relevant studies. In addition, gray literature (the first 10 pages of Google Scholar) was searched using phrases for additional studies.

### 4.4. Search Strategy 

The search strategy aimed to identify published studies that address the objective. An initial search of PubMed and Google Scholar was undertaken to identify articles on the topic. The text words contained in the titles and abstracts of relevant articles, and the index terms used to describe the articles, were used to develop a full search strategy for PubMed, Embase, Scopus, and CINAHL. The search strategy, including all identified keywords and index terms, was adapted for each included database. Two reviewers (SA and RA) conducted the search. Details of search strategies used are provided in the Supplementary File.

### 4.5. Data Management 

The bibliographic details of the identified studies were managed using EndNote20 (Clarivate Analytics, Philadelphia, PA, USA) to remove duplicates. The remaining records were imported into Rayyan.AI (Rayyan Systems Inc., Cambridge, MA, USA) [56] to be screened for eligibility based on predefined inclusion and exclusion criteria. A shared cloud-based folder (Microsoft OneDrive (Microsoft Corporation, Redmond, WA, USA)) was used for collaboration among the authors. All data was backed up regularly, and access was restricted to the authors.

### 4.6. Selection Process

Two reviewers (SA and RA) independently screened the titles and abstracts of identified studies to assess their eligibility based on the predefined inclusion criteria. UA verified the results of titles and abstracts screening and resolved disagreement between the independent reviewers. Full-text articles of potentially relevant studies were retrieved and evaluated by all reviewers. Discrepancies between the reviewers were resolved through discussion and consensus.

### 4.7. Data Collection Process

A data extraction form was created by the authors and included relevant details regarding the study characteristics, patients’ demographics, and antibiotics usage details. Data extraction was performed independently by two reviewers (SA and RA) using a pre-designed and piloted data extraction form to ensure consistency and accuracy. The extracted data was reviewed by the third reviewer (UA) for accuracy, and to resolve any discrepancies. Data extraction forms were created and stored electronically using Excel to ensure consistency.

### 4.8. Data Items

Data collection included the following:

- Study characteristics: Author’s name, year of publication, country, study design, sample size.
- Participants’ characteristics including age, sex, comorbidities, dialysis indication, type of dialysis, and type of infection.
- Antibiotic prescribing details including types, dosing, indication, route of administration, and duration, and the rate and types of inappropriate antibiotic use among the patients. In addition, the appropriateness assessment tool used was extracted. Pre-planned data assumptions include that studies may vary in the definitions of “inappropriate use,” and standardizations will be applied where possible to align these differences across studies.

### 4.9. Outcomes and Prioritization

- Primary outcome: Rate of antibiotics prescribing, types, dosing, indication, route of administration, duration in patients undergoing dialysis across different healthcare settings.
- Secondary outcome: Prevalence and types of inappropriate antibiotics prescribing in patients undergoing dialysis across different healthcare settings.

### 4.10. Risk of Bias in Individual Studies

The risk of bias in individual studies was assessed using the Joanna Briggs Institute Critical (JBI) Appraisal tools. Bias assessment was performed at both the study and outcome levels, assessment focused on biases related to selection, confounding, and measurement. The results of the risk of bias assessment will inform the overall synthesis of the data and will be considered when interpreting the results. This was performed independently by two reviewers (SA and RA). Any discrepancies between reviewers during the process were discussed and resolved with a third reviewer (UA). No study was excluded based on the risk of bias.

### 4.11. Data Synthesis 

Data was synthesized qualitatively through percentages and frequencies. The data synthesized are presented in graphical, diagrammatic, and tabular forms. A descriptive summary accompanied the tabulated and charted results and described how the results relate to the review’s objectives. Quantitative analysis (meta-analysis) was not performed due to variations in the outcomes’ definitions.

## 5. Conclusions

The prevalence of antibiotic use among dialysis patients is relatively higher than hospitalized patients in general, with a considerably large portion of broad-spectrum antibiotics with high propensity for resistance. Up to two-thirds of antibiotics prescribed among dialysis patients are adjudged to be inappropriate, mainly due to dosing, indication, and choice/spectrum of activity concerns. Therefore, an antimicrobial stewardship program is recommended to reduce excessive and inappropriate use of antibiotics in dialysis population.

## Figures and Tables

**Table 1:** Characteristics of studies included in this systematic review.

Author and Year	Country	Study Design	Sample Size	Population	Outcome	Age (Mean ± SD)	Female Gender (%)	Dialysis Type	Dialysis Indication
Al Himali 2022 [26]	Oman	Retrospective cohort	*n* = 287	Adult inpatients who received IHD or CVVHD who were admitted under any medical specialty and received at least one antibiotic	Antibiotic prescribing pattern and appropriateness	58 ± 17	107/287 (37.3%)	IHD and (CVVH)	AKI 86/287 (30.0%) CKD 201/287 (70.0%)
Manley 2002 [16]	USA	Retrospective cohort	*n* = 161	Adult patients who received dialysis at the outpatient hemodialysis center	Antibiotic prescribing pattern and appropriateness	59.98 ± 14.65 ^§^	79/161 (49%)	HD	ESRD (100%)
Snyder 2013 [17]	USA	Ambispective cohort	*n* = 278	Adult patients who received dialysis at either of the outpatient hemodialysis centers	Antibiotic prescribing pattern and appropriateness	66.7 ± 15.5	134/278 (48.2%) ^¶^	HD	ESRD (100%)
Zvonar 2008 [25]	Canada	Retrospective cohort	*n* = 105	Chronic HD patients who received vancomycin at the outpatient hemodialysis center	Antibiotic prescribing pattern and appropriateness	NR	44/105 (41.9%) ^¶^	HD	ESRD (100%)
Udani 2013 [18]	USA	Retrospective cohort	*n* = 723	Adult patients who received either IHD or CRRT in the ICU	Antibiotic prescribing pattern and appropriateness	57.33 ± 14.87 ^§^	230/549 (42%) ^¶^	IHD and (CVVH)	AKI 323/723 (58.8%) ESRD 226/723 (41.2%)
Fedorenko 2014 [19]	USA	Retrospective cohort	*n* = 42	Adult patients receiving CVVHD with a minimum of 24 h of concomitant therapy with one or more IV study antimicrobials *	Antibiotic prescribing pattern and appropriateness	58.4 ± 14.1	22/42 (52.3%) ^¶^	IHD and CVVHD	CKD 6/56 (14%) ^¶^ AKI 36/56 (86%)
Harris 2013 [20]	USA	Retrospective cohort	*n* = 87	Adult patients with ESRD or AKI who received SLED and at least one of the study antibiotics †	Antibiotic prescribing pattern and appropriateness	54 ± 14	35/87 (40%)	SLED	AKI 61/87 (70%) ESRD 8/87 (9%) Unknown 18/87 (21%)
Keough 2018 [21]	USA	Retrospective cohort	*n* = 89	Adult patients admitted to any one of the four adult acute care hospitals with either AKI or ESRD who received SLED for at least two sessions and at least one of the select study antibiotics for at least 5 days ^‡^	Antibiotic prescribing pattern and appropriateness	57 ± 14	21/87 (41%)	SLED	AKI 35/89 (69%) ESRD 16/89 (31%)
Coritsidis 2021 [22]	USA	Retrospective case-control	*n* = 18,402	Adult patients with ESRD on HD and non-ESRD patients with at least one oral outpatient antibiotic prescription	Antibiotic prescribing pattern	64.80 ± 14.80	8330/18,402 (45.30%)	HD	ESRD (100%)
Sivarajahkuma 2022 [24]	Canada	Retrospective case series	*n* = 53	Adult patients who were receiving HD at the outpatient study unit, for whom at least one oral or IV antimicrobial was prescribed by a hospital or community prescriber	Antibiotic prescribing pattern	61 ± 15	25/53 (47%)	HD	ESRD (100%)
Hui 2017 [15]	Australia	Prospective cohort	*n* = 114	Adult patients receiving IHD in either inpatient or outpatient study units	Antibiotic prescribing pattern and appropriateness	63.27 ± 18.17 ^§^	46/114 (40%)	IHD	ESRD (100%)
Saradhi 2020 [27]	India	Prospective cohort	*n* = 110	Adult patients on IHD who have developed infections or were on antibiotics in either inpatient or outpatient study units	Antibiotic prescribing pattern and appropriateness	49 ± 18	11/110 (37%)	IHD	ESRD (100%)
Green 2000 [23]	USA	Prospective cohort	*n* = 103	All patients admitted to the study unit who required IHD	Antibiotic prescribing pattern and appropriateness	56 ± 18	52/103 (50.5%)	IHD	ESRD (100%)

* Antimicrobials were limited to those listed in the institutional CVVHD dosing guidelines requiring renal dose adjustment. † The antibiotics of interest included cefepime, daptomycin, doripenem, gentamicin, imipenem-cilastatin, linezolid, meropenem, piperacillin-tazobactam, tobramycin and vancomycin. ^‡^ The antibiotics of interest included cefepime, daptomycin, meropenem, piperacillin/tazobactam, and vancomycin. ^§^ Calculated using a validated calculator. ^¶^ Not reported by article, calculated by authors based on information provided. Abbreviations: ESRD end-stage renal disease, AKI acute kidney injury, IHD intermittent hemodialysis, CRRT continuous renal replacement therapy, CVVHD continuous venovenous hemodialysis, SLED sustained low efficiency hemodialysis, Abx antibiotics.

**Table 2:** Types and rates of inappropriate antibiotics prescribing among dialysis patients.

Variable	Al Himali et al., 2022 [26]	Manley et al., 2002 [16]	Snyder et al., 2013 [17]	Zvonar et al., 2008 [25]	Udani et al., 2013 [18]	Fedorenko et al., 2014 [19]	Harris et al., 2013 [20]	Keough et al., 2018 [21]	Coritsidis et al., 2021 [22]	Sivarajahkuma et al., 2022 [24]	Hui et al., 2017 [15]	Saradhi et al., 2020 [27]	Green et al., 2000 [23]
Assessment tool	International guidelines	-	National guidelines	International guidelines + ID specialist	International guidelines + expert opinion (nephrologists and critical care pharmacist)	International guidelines	Literature-based recommendations	Literature-based recommendation	-	-	National and international guidelines + renal drug handbook + expert opinion	Micromedex software	International guidelines
Types of inappropriate use	Frequency (%)												
Overall inappropriateness	204/691 (30%)	-	276/926 (29.8%)	Empiric: 17/163 (10%) Definitive: 53/163 (33%)	564/1761 (32.03%) *	41/182 (22.5%) *	66/115 (57.4%) *	147/317 (46.4%) *	-	-	55/224 (24.5%)	23/35 (65.7%)	33/164 (20%)
Breakdown of types of inappropriateness													
Dose	97/204 (47.5%)	-	-		239/564 (42.3%)	41/41 (100%)	66/66 (100%)	125/147 (85%)	-	-	14/55 (25.5%)	21/23 (91.3%) *	-
Interval	107/204 (52.5%)	-	-		325/564 (57.6%)	-	-	22/147 (15%)	-	-	29/55 (52.7%)	2/23 (8.7%) *	-
Duration	-	-	-		-	-	-	-	-	-	6/55 (10.9%)	-	-
Spectrum/choice	-	-	74/276 (26.8%)		-	-	-	-	-	-	13/55 (23.6%)	-	23/33 (69.7%)
Indication	-	-	204/276 (73.9%)		-	-	-	-	-	-	3/55 (5.5%)	-	10/33 (30.3%)

* Calculated by authors based on information available in the studies.

## Data Availability

The data presented in the study are contained within the article or Supplementary Materials. Further inquiries can be directed to the corresponding author.

## Supplementary Materials

The following supporting information can be downloaded at: https://www.mdpi.com/article/10.3390/antibiotics14101049/s1, Table S1: PubMed Database Search Strategy, Table S2: Embase Database Search Strategy, Table S3: Scopus Database Search Strategy, Table S4: CINHAL Database Search Strategy
