# Supplementary material for: Prevalence and Types of Inappropriate Antibiotics Prescribing Among Dialysis Patients: A Systematic Review
Source: Antibiotics (Basel). 2025 Oct 20;14(10):1049. doi: 10.3390/antibiotics14101049 (PMC12562102; doi:10.3390/antibiotics14101049)
Supplement: Supplementary file 1 [file antibiotics-14-01049-s001.zip › antibiotics-3869348-supplementary.pdf]

antibiotics

Systematic Review

Prevalence and Types of Inappropriate Antibiotics Prescribing Among Dialysis Patients: A Systematic Review

Sara Abul-Ola [1], Reem Alenany [1] and Usman Abubakar [2,*]

[1] College of Pharmacy, QU Health, Qatar University, Doha P.O. Box 2713, Qatar; sa1801871@student.qu.edu.qa (S.A.-O.); ra1702907@student.qu.edu.qa (R.A.)
[2] Department of Clinical Pharmacy and Practice, College of Pharmacy, QU Health, Qatar University, Doha P.O. Box 2713, Qatar
* Correspondence: usman.abubakar@qu.edu.qa

antibiotics MDPI

Table S1: PubMed Database Search Strategy

Concepts	Sub-terms	Search options	Number of hits
**1. Dialysis**	1.1 "renal replacement therapy"	MeSH term	241,304 results
	1.2 "renal dialysis"	Title/abstract	2,393 results
	1.3 "hemodialysis"	Title/abstract	76,993 results
	1.4 "haemodialysis"	Title/abstract	17,398 results
	1.5 "hemofiltration"	Title/abstract	3,859 results
	1.6 "haemofiltration"	Title/abstract	1,015 results
	1.7 "hemodiafiltration"	Title/abstract	2,951 results
	1.8 "haemodiafiltration"	Title/abstract	679 results
	1.9 "hemoperfusion"	Title/abstract	2,682 results
	1.10 "haemoperfusion"	Title/abstract	483c results
	1.11 "continuous renal replacement therapy"	Title/abstract	4,169 results
	1.12 "crrt"	Title/abstract	2,879 results
	1.13 "hybrid renal replacement therapy"	Title/abstract	2 results
	1.14 "prolonged intermittent renal replacement therapy"	Title/abstract	55 results
	1.15 "sustained low efficiency dialysis"	Title/abstract	187 results
	1.16 "peritoneal dialysis"	Title/abstract	29,973 results
	1.17 "intermittent hemodialysis"	Title/abstract	1,239 results
	1.18 "intermittent haemodialysis"	Title/abstract	382 results
	1.19 "dialysis"	Title/abstract	130,909 results
	1.20 "intermittent renal replacement therapy"	Title/abstract	130 results
	Total 1 Combine with OR		314,347 results
	Total 1 Combine with OR, Filter: English, Human		234,929 results

**2. Anti-bacterial**	2.1 "anti-bacterial agents"	MeSH term	475,346 results
	2.2 "anti bacterial agents"	Text Word	427,690 results
	2.3 "antibacterial agents"	Title/Abstract	9,969 results
	2.4 "antibacterial agents"	Title	2,047 results
	2.5 "antibiotics"	Title/Abstract	274,357 results
	2.6 "antibiotics"	Title	51,174 results
	2.7 "antibacterial drugs"	Title/Abstract	2,902 results
	2.8 "antibacterial drugs"	Title	493 results
	2.9 "antibacterial medications"	Title/Abstract	71 results
	2.10 "antibacterial medications"	Title	5 results
	Total 2 Combine with OR		614,609 results
	Total 2 Combine with OR, Filter: English, Human		320,056 results
**3. Prescribing Patterns**	3.1 "drug utilization review"	MeSH Terms	7,747 results
	3.2 "drug utilization review"	Text Word	4,154 results
	3.3 "drug utilisation review"	Text word	28 results
	3.4 "drug utilization evaluation"	Text Word	183 results
	3.5 "drug utilisation evaluation"	Text word	6 results
	3.6 "prescribing patterns"	Text Word	4,169 results
	3.7 "utilization patterns"	Text Word	3,174 results
	3.8 "utilisation patterns"	Text Word	297 results
	3.9 "utilization review"	Text Word	12,703 results
	3.10 "utilisation review"	Text Word	76 results
	3.11 "appropriateness"	Text Word	28,527 results
	3.12 "drug use evaluation"	Text Word	216 results
	3.13 "drug utilization"	MeSH Terms	28,787 results

	3.14 “drug utilization”	Text Word	27,422 results
	3.15 “drug utilization”	Text Word	555 results
	3.16 “inappropriate prescribing”	Text Word	6,193 results
	3.17 “inappropriate prescribing”	MeSH Terms	4,963 results
	3.18 “guideline adherence”	Text Word	37,529 results
	3.19 “guideline adherence”	MeSH Terms	35,914 results
	3.20 “practice patterns”	Text Word	81,534 results
	3.21 “medication utilization review”	Text Word	6,345 results
	Total 3 Combine with OR		179,927 results
	Total 3 Combine with OR, Filter: English, Human		146,781 results
**Total**	Total 1 AND Total 2 AND Total 3		82 articles

Table S2: Embase Database Search Strategy

Concepts	Sub-terms	Search options	Number of hits
**1. Dialysis**	1.1 'renal replacement therapy'/syn OR 'renal replacement therapy'	Explosion and synonyms	267,166 results
	Total 1:		267,166 results
**2. Antibiotics**	2.1 'antibiotic agent'/syn OR 'antibiotic agent'	Explosion and synonyms	2,311,054 results
	Total 2:		2,311,054 results
**3. Prescribing patterns**	3.1 'drug utilization'/syn OR 'drug utilization'	Explosion and synonyms	30,063 results
	3.2 'drug utilization review'/syn OR 'drug utilization review'	Explosion and synonyms	20,222 results
	3.3 'appropriateness':ab,ti	Title/abstract	40,041results
	3.4 'prescribing error'/syn OR 'prescribing error'	Title/abstract	12,524 results
	3.5 'protocol compliance'/syn OR 'protocol compliance'	Explosion and synonyms	28,413results
	3.6 'prescribing practice'/syn OR 'prescribing practice'	Explosion and synonyms	10,153 results
	Total 3: combine with OR		129,935 results
Total	Total 1 AND Total 2 AND Total 3		325 results

Table S3: Scopus Database Search Strategy

Concepts	Sub-terms	Search options	Number of hits
**1. Dialysis**<br>Limit to English, article	1.1 {Renal replacement therapy}	Title, abstract, keywords	63,819 results
	1.2 {renal dialysis}	Title, abstract, keywords	90,374 results
	1.3 {kidney dialysis}	Title, abstract, keywords	444 results
	1.4 (hemodialysis)	Title, abstract, keywords	176,761 results
	1.5 (hemofiltration)	Title, abstract, keywords	12,063 results
	1.6 (hemodiafiltration)	Title, abstract, keywords	7,355 results
	1.7 (hemoperfusion)	Title, abstract, keywords	6,851 results
	1.8 {continuous renal replacement therapy}	Title, abstract, keywords	8,803 results
	1.9 (crrt)	Title, abstract, keywords	3,279 results
	1.10 {hybrid renal replacement therapy}	Title, abstract, keywords	35 results
	1.11 {prolonged intermittent renal replacement therapy}	Title, abstract, keywords	71 results
	1.12 {sustained low efficiency dialysis}	Title, abstract, keywords	115 results
	1.13 {peritoneal dialysis}	Title, abstract, keywords	52,364 results
	1.14 {intermittent hemodialysis}	Title, abstract, keywords	1,466 results
	1.15 {intermittent haemodialysis}	Title, abstract, keywords	439 results
	1.16 (dialysis)	Title, abstract, keywords	245,196 results
	1.17 {intermittent dialysis}	Title, abstract, keywords	221 results
	1.18 "intermittent renal replacement therapy"	Title, abstract, keywords	230 results
	Total 3: combine with OR, limit English, article		227,002 results
	Total 3: combine with OR		340,363 results
**2. Antibiotics**	2.1 "antibacterial agents"	Title, abstract, keywords	24,969 results
	2.2 "anti-bacterial agents"	Title, abstract, keywords	315,807 results
	2.3 "antibiotic"	Title, abstract, keywords	1,073,052 results
	2.4 "anti-biotic"	Title, abstract, keywords	317 results
	2.5 "antibacterial drugs"	Title, abstract, keywords	7,511 results
	2.6 "anti-bacterial drugs"	Title, abstract, keywords	219 results

	2.7 "antibacterial agent"	Title, abstract, keywords	24,969 results
	2.8 "anti-bacterial agent"	Title, abstract, keywords	315,807 results
	2.9 "antibacterial medication"	Title, abstract, keywords	202 results
	2.10 "anti-bacterial medication"	Title, abstract, keywords	9 results
	Total 2: combine with OR		1,188,044 results
	Total 2: combine with OR, limit English, article		806,424 results
**3.**	3.1 "drug utilization review"	Title, abstract, keywords	5,147 results
	3.2 “drug utilization evaluation”	Title, abstract, keywords	341 results
	3.3 “prescribing patterns”	Title, abstract, keywords	5,489 results
	3.4 “utilization patterns”	Title, abstract, keywords	6,258 results
	3.5 “utilization review”	Title, abstract, keywords	79,143 results
	3.6 "utilization evaluation"	Title, abstract, keywords	578 results
	3.7 “appropriateness”	Title, abstract, keywords	56,894 results
	3.8 “drug use evaluation”	Title, abstract, keywords	411 results
	3.9 “drug utilization”	Title, abstract, keywords	39,537 results
	3.10 drug use review	Title, abstract, keywords	167 results
	3.11 Drug utilization pattern	Title, abstract, keywords	410 results
	3.12 Drug use pattern	Title, abstract, keywords	1,079 results
	3.13 “inappropriate prescribing”	Title, abstract, keywords	8,317 results
	3.14 “guideline adherence”	Title, abstract, keywords	34,577 results
	3.15 “practice patterns”	Title, abstract, keywords	75,213 results
	3.16 Medication utilization review	Title, abstract, keywords	3 results
	3.17 Medication utilization evaluation	Title, abstract, keywords	10 results
	3.18 Medication use review	Title, abstract, keywords	38 results
	3.19 Medication use evaluation	Title, abstract, keywords	200 results
	Total 3: combine with OR		276,852 results
	Total 3: combine with OR, limit English, article		195,177 results
**Total**	Total 1 AND Total 2 AND Total 3		184 results

Table S4: CINHAL Database Search Strategy

Concepts	Sub-terms	Search options	Number of hits
**1. Dialysis**	1.1 Renal Replacement Therapy+	Subject heading (MH)	38,742 results
	1.2 Continuous Renal Replacement Therapy+	Subject heading (MH)	1,489 results
	1.3 Hemodialysis+	Subject heading (MH)	19,518 results
	1.4 Hemofiltration+	Subject heading (MH)	1,276 results
	1.5 Home Dialysis	Subject heading (MH)	1,057 results
	1.6 Peritoneal Dialysis+	Subject heading (MH)	4,464 results
	1.7 Dialysis+	Subject heading (MH)	27,754 results
	1.8 Continuous Venovenous Hemodialysis	Subject heading (MH)	50 results
	1.9 Continuous Arteriovenous Hemodialysis	Subject heading (MH)	13 results
	1.10 Dialysis	Keyword	37,147 results
	1.11 Hemodialysis	Keyword	25,143 results
	1.12 Haemodialysis	Keyword	14,933 results
	1.13 Intermittent Dialysis	Keyword	16 results
	Total 1: combined with OR, Limiters: English Language; Human		22,610 results
**2. Antibiotics**	1.1 Antibiotics+	Subject heading (MH)	93,567 results
	1.2 Antibacterial	Keyword	8,073 results
	1.3 Antibacterial drug	Keyword	29,111 results
	1.4 Antibacterial drugs	Keyword	186 results
	1.5 Anti-bacterial agent	Keyword	29,050 results
	1.6 Anti-bacterial agents	Keyword	29,053 results
	1.7 Antibacterial agent	Keyword	29,260 results
	1.8 Antibacterial agents	Keyword	29,393 results
	1.9 Antibacterial medication	Keyword	7 results
	1.10 Antibacterial medications	Keyword	11 results
	1.11 Anti-bacterial medication	Keyword	4,136 results
	1.12 Anti-bacterial medications	Keyword	3,986 results

	Total 2: combined with OR, Limiters: English Language; Human		35,706 results
**3. Prescribing patterns**	1.1 Utilization Review+	Subject heading (MH)	13,659 results
	1.2 Prescribing Patterns	Subject heading (MH)	10,412 results
	1.3 Medication Errors	Subject heading (MH)	15,358 results
	1.4 Inappropriate Prescribing	Subject heading (MH)	3,818 results
	1.5 Medication Review	Subject heading (MH)	240 results
	1.6 Guideline Adherence	Subject heading (MH)	18,241 results
	1.7 Medication utilization review	Keyword	44,225 results
	1.8 Prescribing error	Keyword	122 results
	1.9 Appropriateness	Keyword	11,593 results
	1.10 Drug Utilization	Keyword	9,722 results
	1.11 Protocol compliance	Keyword	8,850 results
	Total 3: combine with OR, Limiters: English Language; Human		33,832 results
Total	Total 1 AND Total 2 AND Total 3, Limiters: English Language; Human		21 results
